# Detection of Merkel cell virus and correlation with histologic presence of Merkel cell carcinoma in sentinel lymph nodes

**DOI:** 10.1038/bjc.2012.73

**Published:** 2012-03-13

**Authors:** M Loyo, J Schussel, E Colantuoni, J Califano, M Brait, S Kang, W M Koch, D Sidransky, W H Westra, J M Taube

**Affiliations:** 1 Division of Head and Neck Cancer Research, Department of Otolaryngolgy Head and Neck Surgery, The Johns Hopkins School of Medicine, 1550 Orleans Street CRBII 5NC, Baltimore, MD 21321, USA; 2 The Johns Hopkins Bloomberg School of Public Health, Baltimore, MD, USA; 3 Department of Dermatology, The Johns Hopkins School of Medicine, Baltimore, MD, USA; 4 Department of Pathology, The Johns Hopkins School of Medicine, Baltimore, MD, USA

**Keywords:** Merkel cell carcinoma, Merkel cell polyomavirus, sentinel lymph node, tumour staging, RT–PCR

## Abstract

**Background:**

Adjuvant treatment can dramatically improve the survival of patients with metastatic Merkel cell carcinoma (MCC), making early, accurate detection of nodal disease critical. The purpose of this study was to correlate Merkel cell virus (MCV) detection with histopathologic disease in sentinel lymph nodes (SLNs) of MCC.

**Methods:**

Merkel cell carcinoma cases with SLN (*n*=25) were compared with negative controls (*n*=27). Viral load was obtained by quantitative polymerase chain reaction (PCR) for regions *VP1* and *LT3* of MCV. Histopathologic disease and viral load were correlated.

**Results:**

Merkel cell virus was detected in 16 out of 17 (94%) of primary MCC (mean viral load (MVL)=1.44 copies per genome). Viral load in the negative controls was <0.01 copies per genome. Merkel cell carcinoma was present in 5 out of 25 (20%) SLN by histopathology, and MCV was detected in 11 out of 25 (44%) MCC SLN (MVL=1.68 copies per genome). In all, 15 out of 25 (60%) SLN showed correlation between histologic and MCV results. In all, 2 out of 25 (8%) samples were histopathologically positive and PCR negative. Of note, 8 out of 25 (32%) samples had detectable MCV without microscopic disease.

**Conclusion:**

Patients with positive SLN for MCV even if negative by histopathology were identified. The application of molecular techniques to detect subhistologic disease in SLN of MCC patients may identify a subset of patients who would benefit from adjuvant nodal treatment.

Merkel cell carcinoma (MCC) is a rare, aggressive neuroendocrine carcinoma of the mechanoreceptors in the skin. It has a high propensity for early, regional lymph node metastases. Merkel cell carcinoma has been associated with a newly described polyoma virus, the Merkel cell polyomavirus (MCV or MCPyV) (Feng *et al*, 2008). Merkel cell virus is detected in as many as 80–90% of MCC studied (Feng *et al*, 2008; Busam *et al*, 2009; Shuda *et al*, 2011). It has also been detected in low levels in normal skin, in other inflammatory and neoplastic cutaneous diseases, and in non-lesional skin from patients with MCC (Dworkin *et al*, 2009; Andres *et al*, 2010; Foulongne *et al*, 2010). Despite the seemingly ubiquitous nature of the virus, a combination of findings implicates the virus in the tumourigenesis of MCC. These include the significantly higher prevalence and viral load of MCV DNA in MCC compared with other diverse benign and malignant human tissue samples (Loyo *et al*, 2010), the epidemiologic association with elderly and immunosuppressed patients (Penn and First, 1999; Engels *et al*, 2002; Rubel *et al*, 2002; Albores-Saavedra *et al*, 2010), the integration of virus before clonal expansion of tumour (Feng *et al*, 2008), the presence of signature viral mutations in tumours (Shuda *et al*, 2009), and the expression of viral oncoproteins such as large and small T antigen (Sastre-Garau *et al*, 2009; Shuda *et al*, 2009, 2011).

Evidence suggests that sentinel lymph node biopsy (SLNB) has both prognostic and therapeutic implications for patients with MCC. Patients with lymph node metastases demonstrate a two- to three-fold higher mortality rate when compared with those without nodal involvement (Shaw and Rumball, 1991; Yiengpruksawan *et al*, 1991). Sentinel lymph node biopsy aids in the detection of microscopic nodal disease, identifying an additional one-third of patients with nodal involvement who would have been understaged by clinical staging or imaging alone (Gupta *et al*, 2006). Such detection is critical as there is a significant survival benefit for patients receiving adjuvant nodal therapy when there is histologic evidence of lymph node involvement (Gupta *et al*, 2006). This finding has been further substantiated in a recent study of 5823 MCC patients using data from the National Cancer Data Base, which demonstrated that node-negative status as demonstrated by pathologic evaluation was a better predictor of survival than node-negative status by clinical nodal evaluation alone (Lemos *et al*, 2010), suggesting that a proportion of patients in the latter group actually had occult microscopic nodal involvement. The difference between the prognostic accuracy of histologic *vs* clinically assessed node status is significant enough that the method of nodal assessment has now been incorporated into the new, consensus staging system for MCC (Lemos *et al*, 2010).

Molecular methods such as the quantitative polymerase chain reaction (PCR) have the potential to further increase sensitivity for detecting nodal disease by identifying submicroscopic tumour deposits. Due to the high prevalence of MCV DNA in MCC, molecular detection of viral DNA may serve as a marker for SLN involvement. The purpose of this study was to determine whether MCV DNA can be detected in the SLNs of patients with MCC and to correlate this finding with the histopathologic demonstration of disease.

## Patients and Methods

### Patient specimens

Study approval was obtained from the Johns Hopkins Institutional Review Board (IRB 00034420). The Johns Hopkins Hospital surgical pathology archives were searched for cases of MCC where an SLN biopsy had been performed (*n*=25). The SLN biopsy protocol consists of an intradermal injection of technectium-99m-labelled sulphur colloid to the primary tumour site about 2 h prior to the surgical procedure to allow for the detection of nodal drainage. Intraoperatively, a *γ* detector is used to plan the surgical incision and locate the SLN. Isosulfan blue dye may also be used at the discretion of the operating surgeon at the time of surgery to aid in identification. Sentinel lymph node was defined as the LN that concentrated the highest radiolabel colloid (‘hottest node’). In cases with multiple lymph nodes designated as ‘sentinel’, the one labelled #1 by the surgeon was studied in an effort to include the node with the highest chance of harbouring metastatic disease and decreasing surgeon variability.

Routine haematoxylin and eosin (H&E) staining was performed on the formalin-fixed, paraffin-embedded SLNs from each patient. In addition, before classifying a case as either positive or negative for MCC, at least one immunostain (AE1/AE3, Cam5.2, CK20, synaptophysin, or chromogranin) was performed to confirm the anticipated paranuclear dot-like pattern in positive cases (shown in Supplementary Figure 1) or to exclude subtle lymph node metastases in cases that were negative by H&E. Tissue blocks from the SLN and the corresponding primary tumour (when available, *n*=17) were selected for microdissection and DNA extraction. Negative lymph nodes from patients with mammary carcinoma (*n*=8) and head and neck squamous cell carcinoma (*n*=19) were used as negative controls for MCV.

### Detection of MCV

Ten consecutive 10 *μ*m thick unstained sections were cut from formalin-fixed, paraffin-embedded blocks for each case. The first and last sections were stained with H&E and evaluated histologically to confirm the persistence of tumour in cases with metastases, and to confirm the absence of tumour in cases without metastases. For all cases with tumour, the tumour persisted on the deepest levels. For cases that had originally been diagnosed as negative for tumour, none revealed carcinoma on deeper sections. DNA was extracted from ∼2 mm diameter areas of tumour involvement in both primary and metastatic MCC, resulting in an average tumour cell burden over 50% in the sampled material. In the cases where the metastatic deposits are small (∼0.2 mm in size), it is estimated that a minimum of 5–10% of the cells harvested were MCC cells. For SLNs that were negative for MCC carcinoma, including the negative controls, benign lymphoid tissue of the same approximate total volume was collected.

DNA was extracted by digestion with proteinase K (50 *μ*l ml^–1^ stock solution, final concentration of 2% Boehringer, Mannheim, Germany) in the presence of 1% SDS at 48°C followed by phenol/chloroform extraction and ethanol precipitation. Extracted DNA was resuspended in LoTE (2.5 mM EDTA, 10 mM Tris–HCL (pH 8)) and stored at −20°C. Samples were diluted to 50 ng *μ*l^–1^ of DNA in each reaction, with a total of 150 ng tested per reaction.

Quantification of MCV was achieved by real-time PCR amplification of viral sequences. Primers and fluorescent probes for regions *VP1* and *LT3* of the MCV were designed (see Supplementary Table 1) and quantitative PCR was performed as previously described (Loyo *et al*, 2010). The primers for *LT3* localise to the first exon of the T antigens and hence are able to detect both small T antigens and large T antigens. Samples were run in duplicate. The *β-actin* gene was used to normalise the levels of DNA and as an internal loading control. Molecular grade water was used as a non-template control. Standard curves were developed by diluting the MCV-positive cell line MKL-1 (kindly provided by Dr Yuan Chang and Dr Patrick Moore at University of Pittsburgh) in 90, 9, 0.9, 0.09, and 0.009 ng amounts (Taqman 7900 HT Applied Biosystems, Carlsbad, CA, USA). The MCV has been previously described in the MCC cell line MKL-1 (Shuda *et al*, 2009). Our calculations are based on the MKL-1 cell line containing two viral copies per genome based on personal communication with Dr Shuda (unpublished data). Viral load is reported as the viral number of copies per human genome. Viral load was calculated by averaging the viral copy number from both MCV genes, *VP1* and *LT3*. In those instances where only one of the two viral genes was detected, the samples were considered positive for the virus, and the viral load reported is the viral copy number for the single gene that amplified. Samples with an undetectable signal were considered to have zero copies. To confirm specific amplification of the primers and probes, randomly selected PCR products were run on an agarose gel, revealing single band products that when sequenced corresponded to the region of interest (Loyo *et al*, 2010).

### Statistical analysis

Data were analysed using Stata v10.0 statistical analyses software (Stata Corporation, College Station, TX, USA). Fisher's exact test was used for categorical variables and a Wilcoxon rank-sum test was used for continuous variables. A Kaplan–Meier survival analysis was performed to demonstrate the impact of MCV detection on progression-free survival of patients with histologically negative SLN. Results were considered significant when *P*-values were ⩽0.05.

## Results

### Patient demographics and clinical outcomes

Merkel cell carcinoma patient demographic information is provided in Table 1. Samples were obtained from 25 patients (9 women and 16 men) treated at our institution between 1996 and 2008, with a median age of 61 years (range: 28–87 years). Primary MCCs were located on the head and neck (*n*=8), upper extremity (*n*=10), lower extremity (*n*=6), and trunk (*n*=1). None of the cases had clinically suspected distant metastases at the time of initial diagnosis or SLNB. The mean follow-up time for all patients was 40 months (range: 2–93 months). Recurrence was seen in 5 of the 25 patients (20%), with one patient demonstrating local, nodal, and distant disease, one with nodal and distant disease, two with nodal disease only, and one with distant disease only. All of the patients with a positive SLNB received adjuvant nodal treatment of either additional surgery or radiotherapy. At last follow-up, 15 patients had no evidence of disease, 2 were alive with disease, 4 died of the disease, and 4 died of other causes.

Negative lymph nodes from 27 patients (13 women and 14 men) treated for either mammary carcinoma or head and neck squamous cell carcinoma were used as negative controls. The median age of this group was 65 years (range: 32–91 years), which was not statistically different from the MCC cohort (*P*>0.05).

### MCV detection in primary MCC

Merkel cell virus was present in 94% (16 out of 17) of the primary tumours tested with a mean viral load (MVL) of 1.40 viral copies per genome (range: 1.27–1.48). *VP1* was present in 15 out of 17 tumours. In cases where virus was detected, the mean copy number was 1.41 viral copies per genome (range: 1.28–1.54). *LT3* was present in 16 out of 17 tumours, with a mean viral copy number of 1.39 viral copies per genome (range: 1.26–1.46) in cases with detectable virus. Table 2 shows the viral copy number of MCV in each individual MCC sample tested. There was no significant difference between the levels of the two different viral genes, *VP1* and *LT3* (*P*>0.05).

### MCV detection in LN of patients with mammary carcinoma or head and neck squamous cell carcinoma

Merkel cell virus was not detected in 22 out of 27 LN, and none of the LN tested as controls contained MCV at levels >0.01 viral copies per genome. For the negative SLN from patients with mammary carcinoma, only 3 out of 9 samples had detectable virus (one sample for *VP1* and *LT3* and two samples for *LT3*). For the negative LN from patients with primary HNSCC (head and neck squamous cell carcinoma), only 2 out of 19 samples had any detectable virus (both for *LT3* and none for *VP1*).

### MCV detection in MCC SLNs

In cases where MCV was detected in the SLN, the MVL was 1.68 viral copies per genome (range: 1.44–1.91). *VP1* was present in 11 out of 25 samples. In the 11 cases with detectable virus, the mean copy number of *VP1* was 1.71 copies per genome (range: 1.44–2.01). In the 10 cases with detectable LT3, the mean copy number was 1.68 copies per genome (range: 1.58–1.82) (Table 2). There was no significant difference between the levels of the two different viral genes (*P*>0.05).

Microscopic deposits of MCC were present in 20% (5 out of 25) of SLN while MCV was detected in 44% (11 out of 25) by quantitative PCR. When the histopathology and the PCR results were compared, 60% (15 out of 25) of the samples demonstrate a correlation, with three samples positive and 12 samples negative by both methods. In all, 8% (2 out of 25) had microscopic nodal disease, but were negative for MCV by PCR. Of these two samples, only one primary tumour was available for analysis and was positive for MCV. Notably, 32% (8 out of 25) of samples had detectable MCV without demonstrable microscopic disease. The viral copy number for each primary MCC and corresponding SLN sample is provided in Table 2.

Demographics such as age at diagnosis, sex, location of primary tumour, T-stage, and the presence of other immunologic disease did not differ by viral status (*P*=0.13, 0.07, 0.82, 0.7, 0.89, respectively). Further, the microscopic size of the tumour deposit did not correlate with virus detection. In this limited sample size, the rate of recurrence and the overall survival for patients with histologically negative/MCV-positive SLN was not significantly different when compared with histologically negative/MCV-negative lymph nodes (*P*=0.78 and *P*=0.45). Please refer to Table 1 for comparison of the demographic, recurrence, and survival data of different histology and viral status groups.

## Discussion

Sentinel lymph node biopsy allows for the detection of small tumour deposits, which are otherwise clinically or radiologically undetectable. Such pathologic staging allows for the early identification of patients who could benefit from additional treatment including complete lymphadenectomy or radiation therapy, while sparing patients with no evidence of disease morbidity associated with these procedures. Sentinel lymph node biopsy is routinely used as a part of staging patients with a variety of solid malignancies (Koops *et al*, 1999; Turner *et al*, 2001; Balch and Cascinelli, 2006; Morton *et al*, 2006) and in recent years has been extended to include patients with MCC.

Patients with MCC are excellent candidates for the SLNB procedure, as MCC has a tendency for early, occult spread to regional lymph nodes. Lymph node metastases are present at the time of presentation in 12–31% of patients and occur in up to 76% of patients throughout the course of the disease (Victor *et al*, 1996; Maza *et al*, 2006). Patients with histologically positive SLN that receive adjuvant nodal therapy demonstrate a markedly improved disease-free survival, that is, 51% at 3 years compared with 0% for those without adjuvant therapy (Gupta *et al*, 2006). Specific indications for SLNB, such as primary tumour size >1 cm, are currently being established (Stokes *et al*, 2009). Given the improved prognostic accuracy and potential subsequent treatment benefits, the use of SLNB for MCC is rapidly becoming the standard of care.

The discovery of MCV, a polyomavirus clonally integrated into a high proportion of MCC tumours, provides us with a new molecular target for the detection of MCC. In the present study, we aimed to compare the presence of microscopic disease in SLN to the presence of MCV as detected by quantitative PCR. In our study, MCV was present in 96% (16 out of 17) of the MCC primary tumours tested. The presence of MCV in MCC continues to be confirmed by different independent research groups (Kassem *et al*, 2008; Becker *et al*, 2009; Garneski *et al*, 2009; Loyo *et al*, 2010). In the original report of the discovery of MCV, the polymoavirus was identified in 80% (8 out of 10) of MCC (Feng *et al*, 2008). Australian MCC have been reported to be positive for MCV in 43% (16 out of 37) and North American in 69% (11 out of 16) (Garneski *et al*, 2009). Merkel cell virus presence in European MCC has been reported in 85% (45 out of 75) (Becker *et al*, 2009). The viral region tested also influences detection, as 92% of MCC are small T-antigen positive and only 75% are large T-antigen positive (Shuda *et al*, 2011). Of note, the quantitative PCR technique used in the present study detects both small and large T antigens and was previously used by our research group in a separate cohort, where we demonstrated MCV in 86% (6 out of 7) of MCCs studied (Loyo *et al*, 2010). This suggests that our PCR technique has similar rates of MCV detection to other investigators, and that the slightly higher rate detected in this study of 96% simply reflects a higher presence of virus in this particular cohort.

Merkel cell virus has also been detected in metastatic MCC lymph nodes. In their original paper describing MCV, Feng *et al* (2008) described the presence of the virus in metastatic MCC. Subsequently, MCV was detected in 46% (7 out of 15) of histopathologically positive nodal metastases and in 100% (1 out of 1) of distant metastasis tested (Becker *et al*, 2009). These findings suggest that if the primary MCC harbours MCV, identification of MCV in SLN could aid in detection of metastasis. In our study, the rate of detection of MCV was higher than the detection of microscopic MCC in the SLN. Merkel cell virus was detected in 44% (11 out of 25) of the SLN while microscopic MCC was present in 20% (5 out of 25). In 32% (8 out of 25) of the samples, MCV could be detected without microscopic MCC.

It is possible that testing for MCV could add to the sensitivity of detection of nodal disease and identify patients that would benefit from additional adjuvant therapy. Due to the sample size, we are unable to assess the clinical significance of either the detection of virus or confirm the impact of microscopic disease. Of special interest are the four patients who died of disease (DOD). The primary tumours from these four patients included two tumours that were MCV positive, one that was MCV negative, and one that was not available for testing. Of the four corresponding SLNs from these patients, one was histology-positive/MCV-positive (matched with its corresponding primary tumour), and three were histology-negative/MCV negative. Histology-negative lymph nodes are an independent predictor of survival in this setting (Carter *et al*, 2009); however, three of our patients with negative lymph nodes DOD. It is possible that this unanticipated finding can be attributable to how the SLNs were originally mapped. One of the patients who had a histology-negative/MCV-negative SLN had a primary in her right nostril requiring multiple resections and reconstructive surgeries. Lymphatic drainage in the head and neck can be complicated, especially for midline lesions, and a singular SLN was taken on the right side of the neck. She never demonstrated locoregional disease, but had a recurrence in the liver with lymphadenopathy in the porta hepatis only 3 years following her original diagnosis. Another patient with histology-negative/MCV-negative SLN demonstrated a presumed second MCC primary, which drained to the same axillary nodal basin. The SLN on the second primary was also histologically negative, but the patient demonstrated positive lymph nodes later that same year and shortly afterwards she also developed liver metastasis. Lastly, the third patient who demonstrated histology-negative/MCV-negative SLN had seven lymph nodes designated as SLN by the surgeon, only one of which was tested for the presence of MCV for this study. The patient developed recurrence in the lymph nodes 2 years after the original diagnosis and then progressed to systemic disease. A larger retrospective trial will be necessary to assess the clinical significance of these findings, and may set the ground for a prospective evaluation of the detection of MCV as a prognostic indicator, and potentially an indication for further surgical or radiation therapy.

The accurate determination of viral loads will be essential if molecular methods for MCV detection are to be used for staging metastatic disease. The mere presence of tumour-associated DNA does not necessary imply subhistologic disease, but could potentially represent cell-free nucleic acids draining into regional lymphatics (Yamamoto *et al*, 1997). Of note, 20% (5 out of 25) of the samples had a primary tumour, which had detectable MCV, but which had a negative SLN, militating against the concept that MCV detected in the nodes is simply a reflection of its presence in the primary tumour. Examining the prevalence of MCV serum antibodies, a large fraction of the population has been exposed to MCV (Carter *et al*, 2009). In fact, low levels of passenger MCV have been detected in other benign and malignant tissues, including other non-melanoma skin cancers and haematolymphoid malignancies (Feng *et al*, 2008; Becker *et al*, 2009; Shuda *et al*, 2009; Loyo *et al*, 2010). These low levels of MCV do not represent cancer-associated virus. In contrast, MCV levels identified in MCC are over 60 times higher than MCV detected in other highest human tissues to date (Loyo *et al*, 2010). This allows for threshold levels to be set to differentiate the more ubiquitous presence of the virus from MCC-associated virus. In our study, the samples used as negative controls were either completely negative or positive at extremely low levels (<0.01 viral copies per genome) for MCV. In contrast, the MVL for MCC was 1.12 viral copies per genome in primary tumours and 1.54 viral copies per genome in SLN. These mean levels are consistent with the report by Shuda *et al* (2009) of an average viral load of 5.2 viral copies per genome in primary MCC (range: 0.8–14.3). Similar copy numbers have been reported by other groups. Katano *et al* (2009) reported an average of 0.127 copies per genome (range: 0.04–0.43), Bhatia *et al* (2010) reported an average of 0.127 copies per genome (range: 0.06–1.2), and Laude *et al* (2010) reported an average of three copies per genome (average 3 × 10^−3^ to 3 × 10^3^).

The range of virus detected in the current study was relatively narrow, which is likely a reflection of the design of our assay. In prior reports, there has been variation in the T antigen chosen for interrogation. Bahatia *et al* tested for small T antigen, while Katano *et al* and Laude *et al* tested for the large T antigen. In our experiment, a viral capsid (VP1) and a common T antigen primer for small and large T antigen (LT3) were used, and provided a high detection rate. The lowest threshold for detection using our methodology is ∼1 viral copy per genome in 150 ng of DNA, which is where the exponential growth of our PCR curve begins (Mackay *et al*, 2002). This is a higher threshold than reported in the aforementioned studies, and is a potential explanation for the comparatively restricted range of detection. Notably, since we may not be able to detect samples with very low copy numbers using our methodology, patients with histologically negative SLN who potentially harbour subhistologic disease, as indicated by MCV detection, may be even more prevalent than the 32% that this study suggests. Exploring different primer/probe sets that provide an extended lower range may be of value for future studies addressing the clinical significance of viral load in subhistologic disease and establishing corresponding threshold values for detection.

Merkel cell virus levels detected in the primary tumours and in the SLNs were very similar (average of 1.12 and 1.54 viral copier per genome). We had hypothesised that the primary tumours would have higher viral copy numbers than the SLN, as SLN often had a lower tumour burden; however, this was not the case. This tight range of copy number may also be related to the threshold of detection of our assay. Another possible explanation would be heterogenous virus expression in tumour cells. In human papilloma virus-associated tumours, different intensities in staining by *in situ* hybridisation have been described between different tumour and within different cells in the same tumour (Park *et al*, 1991; Evans *et al*, 2002). One might be surprised that the levels of the virus are the same in tumours and in SLN. We are hypothesising that perhaps the virus-positive cells are more prone to metastasis and hence the population in the SLN is more homogenous and virus positive. If this in fact is true, detecting the virus is more important for staging, as virus-positive cells would be more prone to metastasis.

When the group with histopathologically positive nodal disease was examined (*n*=5), three cases were MCV positive and two cases were MCV negative. One possible explanation for the lack of correlation for the histopathology positive/MCV negative cases is that the primary MCC is negative for MCV. Of these two cases, only one case had primary tumour available for testing, and it was positive for the virus. False negatives, such as this case, could also occur if the sample had a mutation of the MCV region being tested; for example, truncating mutations of the T antigen have been described upon viral integration in MCC (Shuda *et al*, 2009). Testing more than two different viral regions could potentially decrease the prevalence of this problem as well as increase sensitivity of viral detection.

Taken together, these results indicate that clinical threshold levels could be established for the detection of MCC-associated MCV. In one-third of the cases studied, we were able to detect patients with SLN that were positive for MCV by quantitative PCR even if negative by histopathology. These cases had an MVL of 1.54 viral copies per genome, which is keeping with the MVL of primary tumours and histologically evident metastasis, suggesting that this finding represents an early, submicroscopic stage of tumour dissemination. A molecular approach for the detection of MCV may thus be used to identify an additional subset of patients what could potentially benefit from adjuvant nodal treatment. The correlation of these findings with clinical outcomes in a larger study population, likely through a consortium, is essential in determining the prognostic significance of this finding and the potential role for molecular techniques in staging patients with MCC.

## Figures and Tables

**Table 1:** Demographic characteristics for Merkel cell carcinoma (MCC) patients provided for the overall cohort as well as by group for histology/MCV viral status

	**Total, *n* (%)**	**Histology negative/MCV negative**	**Histology negative/MCV positive**	**Histology positive/MCV negative**	**Histology positive/MCV positive**	***P*-value**
Number of patients (*n*)	25	12	8	2	3	
						
Age, median in years	61	67	57	56	68	0.13
						
*Sex*						
Male	16 (64%)	9	6	1	0	0.07
Female	9 (36%)	3	2	1	3	
						
*Location*						
Upper extremity	10 (40%)	4	4	0	2	0.82
Head	8 (32%)	3	3	1	1	
Lower extremity	6 (24%)	4	1	1	0	
Trunk	1 (4%)	1	0	0	0	
						
*T-stage*						
1	11 (44%)	4	4	2	1	0.7
2	2 (8%)	1	0	0	1	
3	0 (0%)	0	0	0	0	
4	2 (8%)	0	1	0	1	
Unknown	10 (40%)	7	3	0	0	
						
Immunologic disease	6 (24%)	4	2	0	0	0.89
						
*Recurrence*	5 (20%)	3	1	0	1	0.78
Local	1 (4%)	0	0	0	1	0.2
Nodal	4 (16%)	2	1	0	1	0.72
Distant	3 (12%)	2	0	0	1	0.4
						
*Clinical status*						0.45
NED	15 (60%)	7	5	1	2	
AWD	2 (8%)	0	1	1	0	
DOD	4 (16%)	3	0	0	1	
DOC	4 (16%)	2	2	0	0	

Abbreviations: AWD=alive with disease; DOC=died of other causes; DOD=died of disease; MCV=Merkel cell virus; NED=no evidence of disease.

*P*-values are based on Fisher's exact test for discrete variables and Wilcoxon rank-sum test for continuous variables.

**Table 2:** Viral copy number of Merkel cell virus (MCV) in primary Merkel cell carcinoma (MCC) and corresponding sentinel lymph nodes (SLNs)

	**Primary MCC**		**SLNs**		
**Sample**	**VP1 (copies per genome)**	**LT3 (copies per genome)**	**Histology MCC**	**VP1 (copies per genome)**	**LT3 (copies per genome)**
1	1.3	1.32	+	1.71	1.71
2	1.41	1.34	+	1.63	1.58
3	1.54	1.38	+	1.73	1.59
4	1.28	1.26	−	1.44	0
5	1.51	1.44	−	1.81	1.63
6	1.4	1.4	−	1.63	1.63
7	1.4	1.45	−	1.7	1.7
8	1.38	1.35	−	1.82	1.78
9	1.42	1.42	−	1.59	1.63
10	1.44	1.43	−	2.01	1.82
11	1.5	1.46	−	0	0
12	1.43	1.42	−	0	0
13	1.36	1.32	−	0	0
14	1.44	1.39	−	0	0
15	1.38	1.39	−	0	0
16	0	1.44	+	0	0
17	0	0	−	0	0
18	NA	NA	−	1.78	1.77
19	NA	NA	+	0	0
20	NA	NA	−	0	0
21	NA	NA	−	0	0
22	NA	NA	−	0	0
23	NA	NA	−	0	0
24	NA	NA	−	0	0
25	NA	NA	−	0	0

Abbreviations: NA=not available; +=positive; −=negative.

The presence of histologically evident disease in the SLN is also provided.
