# Supplementary figures and images for: Detection of Merkel cell virus and correlation with histologic presence of Merkel cell carcinoma in sentinel lymph nodes
Source: Br J Cancer. 2012 Mar 13;106(7):1314–9. doi: 10.1038/bjc.2012.73 (PMC3314790; doi:10.1038/bjc.2012.73)

## Slide 1
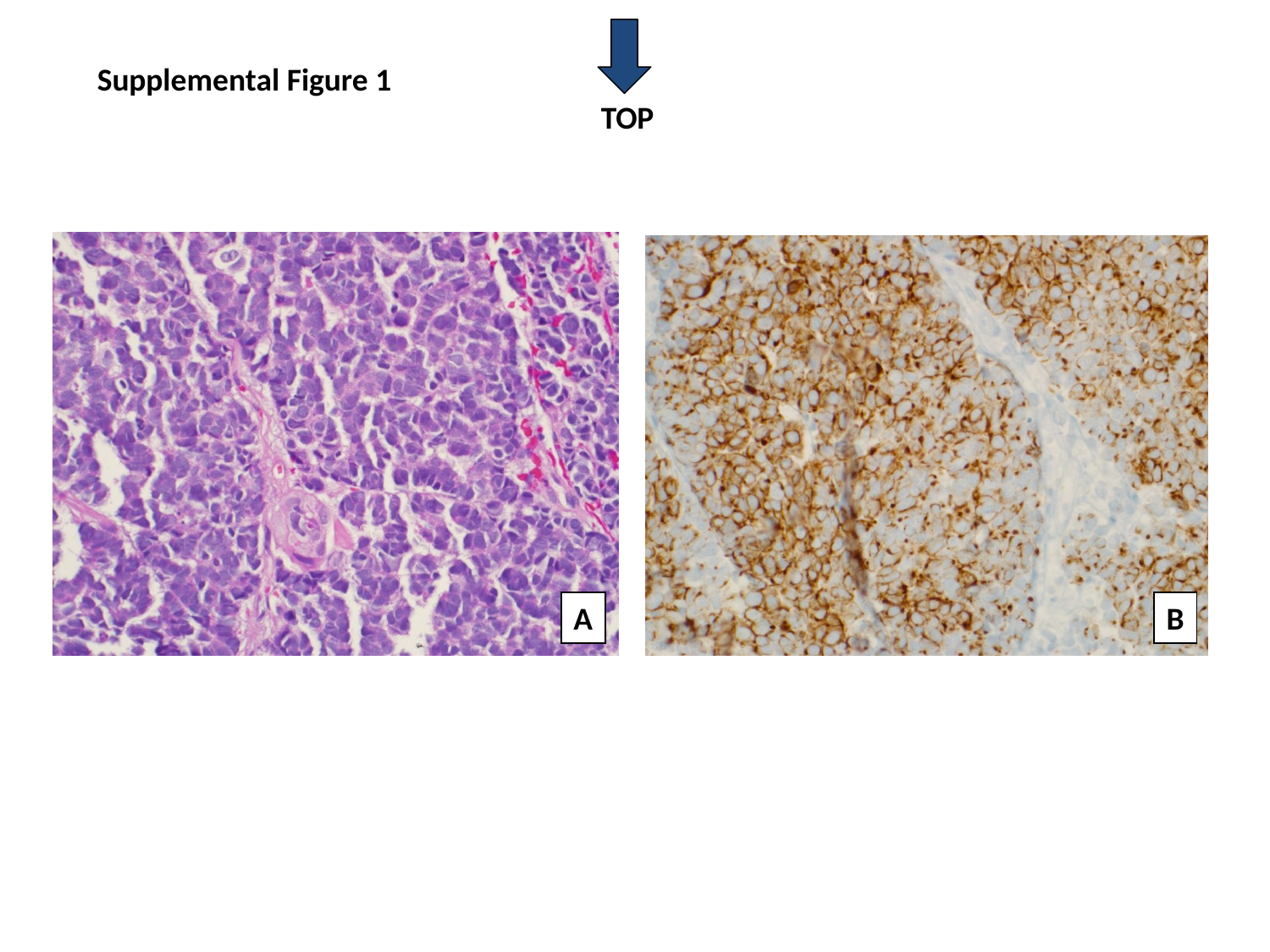

Supplemental Figure 1
 TOP
A
B

Supplement: Supplementary Figure 1 [file bjc201273x1.ppt]
