# Supplementary material for: Detection of Merkel cell virus and correlation with histologic presence of Merkel cell carcinoma in sentinel lymph nodes
Source: Br J Cancer. 2012 Mar 13;106(7):1314–9. doi: 10.1038/bjc.2012.73 (PMC3314790; doi:10.1038/bjc.2012.73)
Supplement: Supplementary Figure Legend [file bjc201273x2.doc]

**Supplemental Figure 1**. A) Metastatic merkel cell carcinoma in a sentinel lymph node, hematoxylin and eosin staining, 400x original magnification. B) Immunohistochemical staining for CK20 in a paranuclear dot-like pattern in the metastatic deposit.
