# Supplementary material for: Detection of Merkel cell virus and correlation with histologic presence of Merkel cell carcinoma in sentinel lymph nodes
Source: Br J Cancer. 2012 Mar 13;106(7):1314–9. doi: 10.1038/bjc.2012.73 (PMC3314790; doi:10.1038/bjc.2012.73)
Supplement: Supplementary Table 1 [file bjc201273x3.doc]

| **Supplemental Table 1**. Primers and fluorescent probes for quantitative PCR detection of MCV. | | | |
| --- | --- | --- | --- |
| Gene | Forward primer (5’-3’) | Probe (5’-3’) | Reverse primer (5’-3’) |
| *VP1* | CCTGATTTTTAGGTGTCATTTT | TTTAGATTACCAGACTGAGTATCCA | GTAAATTACCATATGTTTGCCA |
|  | (3790–3811) | (3867–3891) | (3926–3950) |
| *LT3* | TAAAGCAAAAAAACTGTCTGACG | CTTGGGAAAGTTTTGACTGGTGGCA | TAGAAAAGGTGCAGATGCAGTA |
|  | (602–625) | (680–705) | (734–756) |
| *Β-actin* | TCACCCACACTGTGCCCATCTACGA | ATGCCCTCCCCCATGCCATCCTGCGT | CAGCGGAACCGCTCATTGCCAATGG |
|  | (390–414) | (432–461) | (496–522) |
|  |  |  |  |
